# Genome Sequence of *Klebsiella pneumoniae* KpQ3, a DHA-1 β-Lactamase-Producing Nosocomial Isolate

**DOI:** 10.1128/genomeA.00167-12

**Published:** 2013-02-21

**Authors:** Raquel Tobes, Francisco M. Codoñer, Elena López-Camacho, Iñigo J. Salanueva, Marina Manrique, Marta Brozynska, Rosa Gómez-Gil, Juan F. Martínez-Blanch, Miguel Álvarez-Tejado, Eduardo Pareja, Jesús Mingorance

**Affiliations:** a Oh no sequences! Research group, Era7 Bioinformatics, Granada, Spain; b Lifesequencing S.L., Parc Cientific Universitat de València, Edificio 2, Biotecnología, C/Catedrático Agustín Escardino, Valencia, Spain; c Servicio de Microbiología, Hospital Universitario La Paz, IdiPaz, Madrid, Spain; d Roche Diagnostics Spain, Barcelona, Spain

## Abstract

*Klebsiella pneumoniae* KpQ3 is a multidrug-resistant isolate obtained from a blood culture of a patient in a burn unit in the Hospital Universitario La Paz (Madrid, Spain) in 2008. The genome contains multiple antibiotic resistance genes, including a plasmid-mediated DHA-1 cephalosporinase gene.

## GENOME ANNOUNCEMENT

*Klebsiella pneumoniae* is a Gram-negative bacillus that belongs to the family *Enterobacteriaceae*. *K. pneumoniae* isolates are mostly commensal and environmental microorganisms but may be opportunistic and nosocomial pathogens that cause a variety of infections, including infections of the urinary and respiratory tracts, bloodstream, and soft tissue (1, 2). Nosocomial isolates of *K. pneumoniae* are frequently multidrug-resistant pathogens with severely reduced treatment options that are an increasing problem in the hospital setting worldwide (3).

*K*. *pneumoniae* KpQ3 was isolated from a blood culture from a burn unit patient in the context of a small outbreak. This organism is resistant to several antibiotics, including most β-lactam and β-lactamase inhibitor/β-lactam combinations, and was found to harbor a DHA-1 β-lactamase gene, coding for an AmpC plasmid-mediated cephalosporinase (4).

To obtain the *K. pneumoniae* KpQ3 genome sequence a shotgun and a paired-end library (8 kb) were generated and sequenced in a Roche 454 GS-FLX+ sequencer, resulting in 631,045 reads (43.27-fold coverage). Assembly was done with Newbler and manually refined. The KpQ3 genome was submitted to GenBank (AMSU00000000) with 73 contigs assembled in 18 scaffolds with a genome global length of 5,401,505 bp. Genome annotation was done using the Bacterial Genome Annotation system BG7 (5) and the functional annotations are available at GenBank.

The entire genome consists of 5,135 genes, including 4,931 protein-coding genes.

It is suspected that the genome consists of four circular replicons, one chromosome, and three plasmids. The chromosome (5,245,407 bp, 57.53% G+C) belongs to sequence type 37 (ST37) (6) and contains 4,785 protein-coding genes, 81 tRNA genes, and 8 copies of the rRNA ribosomal operons.

The largest plasmid is 81,155 bp long (53.08% G+C), contains 80 protein-coding genes, including five aminoglycoside resistance genes and a VirB type IV secretion system (T4SS) operon, and is closely related to the *Salmonella enterica* plasmid pCVM19633_110 (GenBank accession number CP001125).

The second putative plasmid is 57,382 bp long (53.96% G+C) and carries 61 protein-coding genes, including the gene for the plasmid-mediated cephalosporinase DHA-1, the gene for OXA-1 β-lactamase, and the gene for a quinolone resistance determinant (QnrB4), among other antibiotic resistance genes.

The third plasmid is a small one, with 4,905 bp (52.62% G+C); it carries 5 protein-coding genes and is very similar to the p58 plasmid from *Escherichia coli* (FN649416) except for the insertion of a DNA methyltransferase gene similar to the *Vibrio* sp. hemagglutinin-associated protein gene.

### Nucleotide sequence accession number. 

The genome of *Klebsiella pneumoniae* KpQ3, with functional annotations, is available in GenBank under accession number AMSU00000000.
